# The effect of grade retention on secondary school performance: Evidence from a natural experiment

**DOI:** 10.1371/journal.pone.0345322

**Published:** 2026-03-17

**Authors:** Sergio Parra-Cely, Bart H. H. Golsteyn, Maria Ferreira-Sequeda

**Affiliations:** 1 School of Economics, Universidad San Francisco de Quito USFQ, Quito, Ecuador; 2 School of Business and Economics, Maastricht University, Maastricht, The Netherlands; 3 Consumer Insights Team, Netflix, Amsterdam, The Netherlands; PLoS ONE, UNITED STATES OF AMERICA

## Abstract

We study the effects of grade retention on secondary school performance using a change in legislation in Colombia. In 2010, the rule that allowed schools to retain a maximum of 5 percent of their students was abolished. We exploit variation in changes in schools’ retention rates across time in a difference-in-differences framework, and find that higher school retention rates improved performance on high-school exit tests scores. While gains in learning did not differ between STEM and non-STEM subjects, increased retention disproportionately benefited non-retained students, students from higher socioeconomic backgrounds, private schools, and schools operating under a full day shift.

## 1. Introduction

Retention in school is common and widespread, but its consequences for school performance are theoretically unclear and empirically diverse. In the United States, around 10 percent of all students are retained between kindergarten and eighth grade. In Germany and France, respectively 9 and 18 percent of all students are retained in primary school [1]. Effects can be expected both for retained and non-retained students. For the retained group, there may be positive effects as repeating a grade can help to acquire basic knowledge needed to perform well later on. But retention may instead also have negative effects on school performance if, for instance, self-esteem and motivation decrease as a result. For non-retained students, the relationship between grade retention and performance works via different mechanisms. In principle, students at the upper end of the ability distribution may learn more and perform better as the level of teaching increases if weaker students in class are retained or if sharing the classroom with fewer low-ability students. A positive effect of retention at the lower end of the ability distribution may be that the threat of being held back can stimulate children to work harder in school. One may also argue that this threat leads to other effects. Belot and Vandenberghe (2014) [2] exploit a law reform to find that an enhanced threat of grade retention does not lead to better medium-term outcomes. But this threat may also have negative consequences, as there is a negative relationship between mental stress and academic performance. Taken together, further empirical research on the effect of retention on school performance is needed as its expected effects are ambiguous from a theoretical point of view.

An important empirical challenge in studying the relationship between grade retention and school performance is that omitted variables may drive the relationship. For instance, high ability children may be less likely to be retained and may also obtain higher school grades. This implies that a naive estimation of the effect of retention on academic achievement may be negatively biased for retained students and positively biased for non-retained students. Moreover, the very nature of grade retention changes class ability composition over time, which implies that retained and non-retained students will differ in their future grade levels, age, group of peers and expected graduation date [3,4].

In this paper, we propose a framework to overcome these endogeneity problems and recover an estimate that comes closer to the causal effect of grade retention on secondary school performance of both retained and non-retained students, accounting for the compositional effects of retention at the school level. To do this, we combine individual data on students’ performance at a nationwide, high-school exit standardized exam with schools’ administrative records on grade promotion and retention. Specifically, we analyze the aggregated net effect of increased retention at the school level on academic performance in 11th grade, the last year in secondary school (nominal age: 16–17), using two administrative datasets from Colombia. The first, provided by the National Inspectorate of Education, includes data for all students in the country on scores from the centralized exam in the last year of secondary education. The second dataset, from the National Bureau of Statistics, contains information on retention rates across all schools and grades in the education system. We are able to link the two datasets using unique school identifiers with a 95% success rate.

To overcome the endogeneity problem before mentioned, we exploit a policy change with respect to retention. From 2002 to 2009, under the automatic promotion policy regime (AUP, hereafter), schools were, by law, not allowed to retain more than 5 percent of their students. In 2010, this directive was abolished and since then, schools are free to decide how many students should repeat a grade. The repeal of the AUP directive increased retention rates dramatically in some schools, while in others it had no effect. We use this information in a difference-in-differences (DID) framework, in which treatment and control groups are defined by changes in retention attributed to the law change. Schools in which the number of retained students (all grades combined) increased more than the median change are labeled the treatment group and those with retention rates below the median, the control group. To ensure treatment and control groups are fairly comparable, we implemented a nearest-neighbor propensity score matching procedure (NN-PSM) to select a sub-sample of schools that, irrespective of their treatment status, could be considered similar to each other during the AUP period in any other observable dimension. We estimate event-study (EE) and two-way fixed effects (TWFE) models to recover the dynamic effects of increased school retention on academic performance, as well as to document heterogeneous effects by exam subjects (i.e., STEM vs. non-STEM), school’s attributes (including students’ socio-demographic composition), and across the test scores distribution. We also conduct several robustness checks to test the validity of the parallel trends assumption, providing reassuring evidence that i) trends in test scores were plausibly similar in control and treated schools before the AUP directive was repealed, and ii) findings remain robust even when we consider sizable violations from the assumed common trends in test scores during the AUP period.

Estimates from our event-study specification indicate that the repeal of the AUP regime had a positive effect on academic performance. Three years after the AUP repeal, treated schools experienced an additional 0.11 standard deviation (SD) increase in their exams scores, relative to schools in the control group. Performance gains were uniform across all exam subjects, as we do not find evidence that positive effects differ between STEM and non-STEM fields. TWFE estimates suggest an average increase of 7% of a SD in exam scores as a consequence of increased grade retention. This effect is equivalent to 0.3 decile improvement in academic performance across the test score distribution, corresponding to a 6% increase from the median performance at baseline.

A key feature of our model is that we analyze the aggregated effects of retention distinguishing between the effects on retained and non-retained students. At the individual level, we do not have information on whether a student has been retained. However, we can identify the effects on retained and non-retained students by i) leveraging variation in AUP (lack of) exposure for different exam cohorts, and ii) exploiting the fact that the high-school exit exam is taken in the third quarter of the academic year, without pupils or schools knowing with certainty which students are effectively graduating from high-school. An important piece of information here is that retention rates at 11th grade (the last year of high-school) remain constant before and after the AUP repeal. Recall that retention rates increased in treatment schools in 2010. Hence, exams cohorts 2007–2010 were not disproportionately exposed to the increased retention policy change during their secondary education whatsoever. This implies that one year later, in 2011, only the students who passed 10th grade in 2010 will be taking the final exam in 11th grade. Therefore, exam scores in 2011 will capture the performance effect that is attributable to non-retained students. One year later, in 2012, students who were (only once) retained in 10th grade in 2010, and students who were promoted from 10th grade in 2011 will be taking the final exit exam. Consequently, exam cohorts from year 2012 onward will capture the retention effect for the mixed group of non-retained and retained students.

In our TWFE regressions, we analyze the effect of the new, free retention policy regime (FRP, hereafter) on exam grades in both years simultaneously, by using the interaction of the treatment status indicator with two variables taking value one if a particular exam cohort was exposed to the FRP regime one or two years prior. Controlling for the non-retained group (the first lag), allows us to identify the retained students within the mixed group (i.e., the second lag gives the effect on the retained and non-retained students minus the effect on the non-retained students). We identify i) the non-retained students with the first lag, and ii) the retained students with the second lag. This way, we exploit idiosyncratic variations in retention rates due to the reform as well as variation in the proportion of repeating students across adjacent exam cohorts within the same schools. Since we have enough data for the years before and after the reform, the compositional effect of retention over time will be accounted for in our difference-in-differences approach, making it possible to identify the net effect of retention on the performance of both retained and non-retained students.

Results from this empirical exercise show that although both groups of students benefited from the AUP repeal, effects for non-retained students were roughly twice as large as those for retained students. For retained students, the gains were driven primarily by positive effects in STEM subjects, especially math, while effects in non-STEM areas were less precise. This pattern suggests that much of the observed improvement in test scores reflects a positive selection effect, with non-retained students benefiting the most. Additional heterogeneity analyses indicate that schools with full-day schedules and privately funded schools reaped the largest gains from increased grade retention. In terms of socioeconomic differences, male students and those from low-educated households benefited disproportionately from the AUP repeal. Mechanism analyses further show that higher retention rates at treated schools significantly affected dropout and transfer rates. We also document variation across the exam score distribution: treated schools were 3 percentage points more likely to place in the top quartile, equivalent to a 7.5 percent standard-deviation effect. However, this result is driven by schools with above-median performance on the high-school exit exam during the social-promotion years.

Taken together, the findings suggest that the performance gains associated with increased retention stem from positive sorting within the school system and from stronger implicit incentives that encouraged students from more advantaged backgrounds to exert additional effort to avoid retention.

Our analysis contributes to the literature in various disciplines that have studied the effects of retention on school performance. Several articles in School Psychology and Sociology of Education analyze the relationship between grade retention and later school performance, mostly reporting this relationship to be negative. McCoy and Reynolds (1999) [5] report that retention has a negative relationship with reading achievement. Jimerson et al. (1997) [6] find no evidence that retention is related to school performance. Jimerson (1999) [7] follows students for 21 years in a longitudinal study to show that retained students have worse educational and employment outcomes in late adolescence. Silberglitt et al. (2006) [8] find that retained students made less educational progress compared to a random group of other students. Stearns et al. (2007) [9] report that students who repeat a grade prior to high school have a higher risk of dropping out of high school than students who are continuously promoted. An important caveat is that these articles report correlations and not causal estimates. Although correlations are informative, important confounders may bias such estimates. As previously explained, we expect a downward (upward) bias for retained (non-retained) students.

There is a small but growing literature that estimates the causal effect of grade retention on subsequent educational outcomes. Hill (2014) [10] investigates the extent to which course repeaters in high school mathematics courses exert negative externalities on their course-mates. Using individual and school-specific course fixed effects to control for ability and course selection, the study shows that increasing the share of repeaters in each course results in a moderate, significant increase in the probability of course failure for first-time course-takers. Results suggest that the negative effect is only evident when the share of repeaters reaches a threshold of 5–10 percent of the total number of course-takers.

The literature reveals that effects of retention are mixed, documenting positive as well as negative estimates. The results depend on the context and age of students. Firstly, some papers study the effects of retention at young ages. Koppensteiner (2014) [11] examines the effect of automatic grade promotion on academic achievement (math scores) at primary school in Brazil. Applying a difference-in-differences approach that exploits variation over time and across schools in the grade promotion regime, the author finds a negative and significant effect of about seven percent of a standard deviation on math test scores. Fruehwirth et al. (2016) [1] evaluate the effect of retention on achievement using data from children in kindergarten. Accounting for dynamic selection into retention, they find that children who are retained in kindergarten would have performed as much as 27 percent higher on math and reading tests in the next year if they had not been retained. Jacob and Lefgren (2004) [12], instead, find positive effects of retention at an early age. They assess the effects of retention in the Chicago Public School system using variation in retention generated by a test-based promotion policy, and find that retention has a modest but positive net impact on test scores for third grade students, while it increases academic achievement for low-achieving third graders. However, they also find that retention appears to have little or no effects for sixth-grade students.

Secondly, some studies have assessed the effects of retention on achievement in high school. A first set of papers reports negative effects. Jacob and Lefgren (2009) [13] show that retention among younger students (sixth grade) does not affect the likelihood of high school completion, but retaining low-achieving eighth grade students in elementary school increases the probability that these students will drop out of high school. Manacorda (2012) [14] studies the effects of retention in secondary junior high school (grades 7–9) in Uruguay on dropout rates and school attainment, exploiting a discontinuity established by a rule of automatic grade failure for pupils with more than three failed subjects at the end of the school year. The analysis reveals that retention increases school dropout and reduces school attainment. While analyses in secondary education focus mostly on dropout rates or completion of school, García-Pérez et al. (2014) [15] measure the effect of grade retention on Spanish students’ PISA math scores at age 15, using the student’s quarter of birth as an instrumental variable. They find that grade retention has a negative impact on educational outcomes. Those who are retained during primary education suffer more than those retained in secondary school.

Contrary to these findings, a second set of papers provides estimates of positive effects of retention in high school. Mahjoub (2017) [16] finds large positive effects of retention on test scores, using quarter of birth as an instrument. The average effect of the treatment on the treated (ATT) ranges between one and one-quarter of a standard deviation of the test scores. Grade repetition in junior high school is also shown to increase the probability of graduation by 2.5 percentage points. Schwerdt et al. (2017) [4] use administrative data from public schools in Florida to study the effects of a policy mandate by which third grade scoring on a statewide assessment test determined students’ retention and remediation. Following a fuzzy regression discontinuity design, they find that the effect of third grade retention and remediation is substantially positive three years after retention: retained students outperform their same-age peers who were promoted by 0.31 standard deviations in reading and by 0.23 standard deviations in math. These same-age positive effects fade out over time, becoming statistically insignificant in both subjects within five years, but retained students continue to outperform their promoted peers when tested in the same grade through grade eight in math and grade ten in reading. In addition, they find that being retained in third grade improves students’ grade point averages (GPAs) and leads them to take fewer remedial courses in high school. Their estimates capture the combined effect of retention and additional remediation measures and may not be directly comparable to those of some previous studies of retention. Eide and Showalter (2001) [17] use an instrumental variable for retention based on exogenous variation across states in kindergarten entry dates to find tentative evidence that retention may benefit students by both lowering dropout rates and raising labor market earnings. They find these effects to be relevant for white students, but not for black students.

A common approach in these studies is that the benefits of retention are evaluated at the margin where retention was increased by the natural experiment. An important issue with this approach is that the estimated benefits may differ at other moments of the ability distribution of students. For low performing students, the benefits of repeating a class may be positive while for high performing students there are probably negative effects. Schools are aware of this and aim to retain students until the marginal student does not benefit from retention.

Our analysis contributes in several ways to the literature studying the effects of retention on educational outcomes. This paper contains the first analysis that highlights that the effects of retention vary across the ability distribution. This result indicates that effects of retention found in one part of the ability distribution cannot be extrapolated to other parts of this distribution. In line with this finding, we show also that the effects differ for retained and non-retained students. We show that increases in retention disproportionally benefit non-retained students. This result indicates that to evaluate the costs or benefits of retention for society, it is important to take the aggregated effects for both retained and non-retained students into consideration.

Our paper provides evidence on the effect of retention for a developing economy using a large administrative dataset. The empirical approach we use to elicit causal effects departs from most other papers in this literature. We exploit the effects of a law change, which enabled schools to retain more children. The analysis developed by [11] comes closest to our setting and approach. He examines the effect of automatic grade promotion on academic achievement (math scores) in primary schools in Brazil using a difference-in-differences approach. Besides that we evaluate effects of retention separately for retained and non-retained students, and that we study the effects in secondary education and not in primary education, our study differs from his in the sense that we can show with several robustness checks that the pre-treatment trends in school performance are common in treatment and control groups, i.e., the key underlying assumption of the difference-in-differences framework. Koppensteiner (2014) [11] does not analyze the commonality of the trends but applies a more static approach by showing instead that school and student characteristics of treatment and control groups tend to be similar before the treatment occurred.

The setup of this paper is as follows. Section 2 summarizes the Colombian context and the educational reform we study. Section 3 describes our main sources of information, sample selection concerns, and the final dataset construction. Section 4 discusses the empirical strategy in detail. Our central findings, including heterogeneity and mechanisms analyses, are presented in Section 5. Section 6 provides several robustness checks that confirm our methodology and results. Finally, Section 7 concludes.

## 2. Institutional background

### The secondary education system in Colombia

Colombia has an eleven-year system of elementary and secondary education, consisting of five years of primary school (1st to 5th grade), four years of lower secondary education (6th to 9th grade) and two years of upper secondary education (10th to 11th grade). Elementary and secondary education in Colombia is offered in two academic calendars: A calendar labeled “A” that runs from February until November, and a calendar “B” from September to June. Most schools (92%) in the country operate in calendar A. Formal education for students in schooling age is also offered in three different daily shifts: a morning schedule (7:00–12:00), an afternoon schedule (13:00–18:00), and a full-day schedule (8:00–17:00). Students opt or are allocated by each school to attend either one of these. Most students in secondary education attend school either in the morning or the afternoon shift (78%). The expected age of school enrollment at 1st grade is six years old. This age is suggested but not mandatory as in Colombia there are no compulsory age-at-school entry laws. Therefore, if children are not retained, they are expected to complete their secondary education at ages 16–17.

The educational system in Colombia is a comprehensive school system with no academic tracking at any grade. The Ministry of Education regulates and monitors all levels of education and national exams for both publicly and privately funded schools. However, at the start of upper secondary education, schools differentiate in the provision of additional courses to complement the compulsory curriculum set by the Ministry of Education. These additional courses are organized in two specialization programs: one is more academic and the other more technical in nature. The academic program provides general education in arts, sciences and humanities, whereas the technical program provides vocational knowledge and practice in technology, craft industry, business, pedagogic training (to prospective elementary school teachers), or agriculture.

Upon completion of the 11th grade of secondary school, all students, regardless of the chosen program, participate in the national standardized exam SABER11, an achievement and competency test that is administered every year by the Ministry of Education’s Colombian Institute for Educational Assessment (*Instituto Colombiano para la Evaluación de la Educación*, ICFES), which acts as the National Inspectorate of Education. This exam is a high-stakes evaluation, required not only to receive the high-school diploma, but also to opt for admission into tertiary education. This test is also widely considered as the reference performance measure to evaluate the quality of secondary education across the country. This exam takes place every year during the third quarter of the academic calendar A, and all students in the country take this exam in the same weekend.

Raw test scores range from 0 to 100 in each subject and they are standardized by subject and year at the national level, so scores are informative about each student’s relative position in the national performance distribution. The SABER11 exam assesses eight subjects. Four fall within the STEM category (mathematics, physics, biology, and chemistry), while the remainder subjects cover non-STEM competencies: language (writing and reading comprehension in Spanish), social sciences (geography and history), philosophy, and English (as a foreign language). The overall exam score corresponds the sum of all eight individual subject scores, weighting in equal importance. With this final score, the Inspectorate of Education ranks students and schools by performance percentiles. To ease comparisons across years and schools, all scores are annually standardized and expressed in standard deviations (SD). Building on existing literature on grade retention, we examine students’ performance in the entire exam, as well as in its STEM and non-STEM components separately.

### The Automatic Promotion Policy Rule (AUP)

In 2002, by mandate of the Ministry of Education (Decree 230 of 2002), schools were each year permitted to retain up to a maximum of five percent of their students. This retention policy was implemented to reduce costs attributed to higher retention rates (i.e., low performance, low motivation, dropouts, etc.) without compromising the quality of education provided by the system. According to this policy mandate, a student should have been retained if at least one of the following three circumstances occurred: i) the student received an unsatisfactory performance evaluation in three or more academic subjects in the current academic year, ii) the student received an unsatisfactory performance evaluation in math and/or language courses during the current and two previous grades, or iii) the student failed to attend at least 25% of all academic activities during the current academic year. However, the AUP rule was enforced at the school level. This means that schools were required to adjust their evaluation standards at the grade and school-levels to comply with the law, forcing them to promote at least 95% of all their students [18].

While the reform was marketed as moderately successful in terms of reducing school dropout, the incentives to underperform at school as perceived by school boards, teachers, and parents, led the Ministry of Education to revoke the AUP Rule (Ministry of Education, Press Release April 17, 2009). In February 2009, the five percent cap in retention rates was replaced by the Ministry of Education through a new regulation mandate (Decree 1290 of 2009). The new decree allowed schools from 2010 onward to retain as many students as they considered necessary, thereby giving them more discretion in their evaluation and promotion procedures.

**Fig 1:**
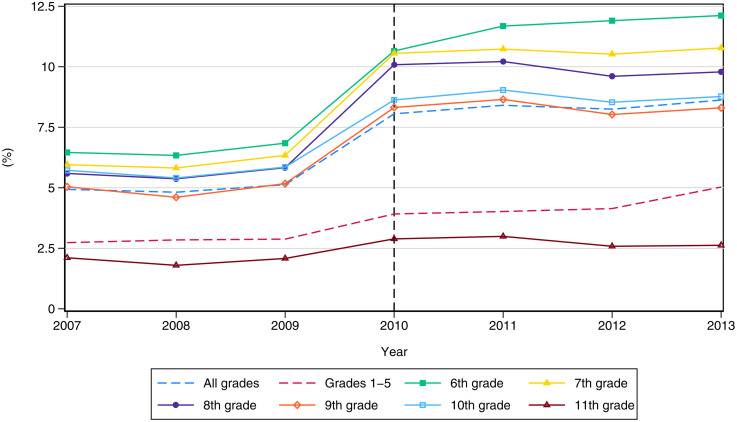
Retention rates per school and grade. Source: Authors’ calculation based on administrative information of the C-600 school census from the National Bureau of Statistics DANE.

Fig 1 displays average retention rates per school and by academic grade during period 2007–2013. Retention rates during the AUP period concentrate around the 5 percent cap, while perfect compliance was not achieved given the Ministry of Education lack of directives on how to legally sanction schools who failed to comply with the AUP policy. Overall, the abolition of the AUP regime increased, on average, retention rates across all schools and grades in the country from 4.9 to 8.3 percent, a 3.4 percentage points (p.p.) difference equivalent to a 70% rise relative to retention levels during the AUP period. Most of this boost in retention is explained by schools being more strict with students during the first three years of lower secondary education (grades 6th to 8th). Retention rates in subsequent years (grades 9th and 10th) exhibit a three p.p. increase relative to baseline values. In contrast, retention rates during primary school, and at the end of secondary education (grade 11th), seem to have been unaltered by the policy change.

## 3. Data overview

### Sources of information

We rely on two administrative datasets for student- and school-level information. The first is the SABER11 high-school exit exam dataset, administered by the National Inspectorate of Education (ICFES). It provides freely downloadable, anonymized individual-level data on test scores, personal characteristics (gender, age, municipality of residence), and socioeconomic attributes (household income bracket, parental education) for the universe of students who took the centralized 11th-grade exam. The dataset also includes basic school characteristics, such as public/private status, academic orientation, academic calendar, and working day-shift type. According to Inspectorate of Education guidelines, test scores are comparable from 2000 to 2013.

The second source of information corresponds to the administrative census of schools reported to the National Bureau of Statistics (*Departamento Administrativo Nacional de Estadística*, DANE). Known as the C-600 census, this dataset provides grade-level academic indicators that all schools in the country are required to report annually. It includes promotion, retention, dropout, and transfer rates, along with school characteristics such as location, student enrollment and class groups, share of students from ethnic minorities, number of teachers with professional degrees, and size of non-academic staff (managerial, general services, and health). We consider the period 2007–2013, as it corresponds to the period in which the C-600 registers are publicly available, comparable across years, and contain school-level identifiers that enable a reliable matching with the SABER11 dataset.

### Sample selection and classification of treated and control schools

Schools responded differently to the new policy, indicating that the effect of grade retention varies across institutions. We classify schools into two groups: the treated group, consisting of schools that increased their retention rates after the law change, and the control group, composed of schools whose retention rates remained relatively stable. Classification is based on the difference in each school’s average retention rate across the two policy regimes, the AUP regime (2007–2009) and the FRP regime (2010–2013). Schools with an above-median increase in retention rates form the treated group, while the remaining schools are assigned to the control group.

**Fig 2:**
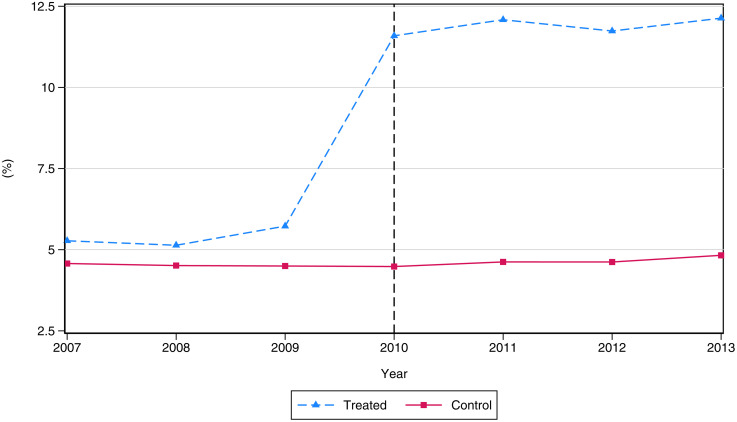
Retention rates per treatment status. This figure shows average school retention rates by treatment status. The dotted line displays statistics for treated schools, while the continuous line shows statistics for control schools. Treated schools are defined as those with above-median retention rate change between the AUP and FRP periods. Statistics are computed using the full sample of schools.

**Fig 3:**
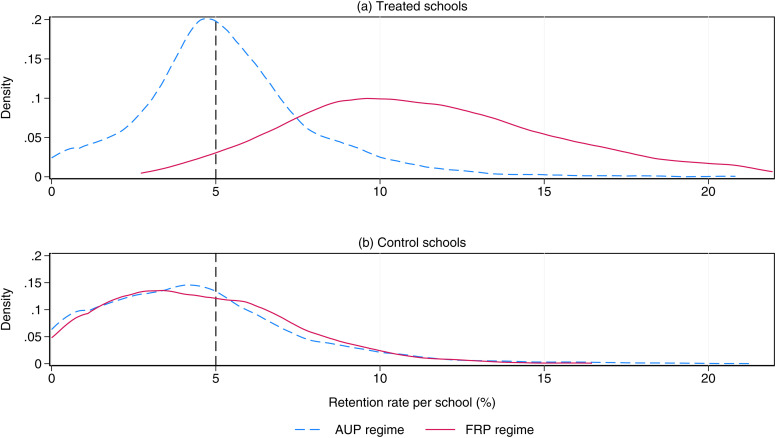
Distributions of school retention rates by treatment status. This figure shows the distribution of the average retention rate by (a) treated and (b) control schools, and by periods of analysis. Dotted lines display distributions during the AUP period, while continuous lines display distributions for the FRP period. The vertical dotted line marks the 5% retention cap. Distributions are computed using the full sample of schools.

Fig 2 displays average school retention rates by treatment classification. During the AUP period (2007–2009), retention rates hovered around the five-percent cap. From 2010 onward, schools in the treated group show a 6.5 p.p. increase in average retention rates relative to control schools, equal to 1.2 times the baseline average. In contrast, control schools experienced only a 0.11 p.p. increase. Comparing the distribution of retention rates across groups, control schools show higher compliance during the AUP regime (Fig 3). After the AUP repeal, 59 percent of control schools continued to retain students below the five-percent cap, compared with only 3 percent of treated schools that did not increase retention.

### Summary statistics

In Table 1, we present the summary statistics of school attributes during the AUP period (2007–2009). Panel A reports mean differences in school characteristics that are available in the C-600 census data. Results for the full sample of schools suggest that treated and control schools dramatically differ in many aspects (columns (1) to (5)). On average, a large proportion of treated schools are urban, privately funded, and operate in the morning shift. Treated schools also seem to offer more variety of academic options for students, have more students enrolled, and also are better equipped in terms of number of classrooms, supporting staff, and teachers with better qualifications.

**Table 1:** Summary statistics of schools during the AUP regime (2007-2010).

	Full sample					Matched sample				
	Treated	Control	Difference			Treated	Control	Difference		
	Mean	Mean	Absolute	P-value	Normalized	Mean	Mean	Absolute	P-value	Normalized
	(1)	(2)	(3)	(4)	(5)	(6)	(7)	(8)	(9)	(10)
Panel A: School attributes from DANE C-600 Census										
Private school (%)	10.40	39.98	-29.58	0.00	-0.51	15.75	16.59	-0.84	0.42	-0.02
Public school (%)	89.60	60.02	29.58	0.00	0.51	84.25	83.41	0.84	0.42	0.02
Rural school (%)	25.96	26.03	-0.07	0.95	-0.00	34.64	31.48	3.15	0.02	0.05
Urban school (%)	74.04	73.97	0.07	0.95	0.00	65.36	68.52	-3.15	0.02	-0.05
Working spell: full day (%)	17.69	35.63	-17.95	0.00	-0.29	23.68	23.71	-0.03	0.98	-0.00
Working spell: morning (%)	60.27	52.19	8.08	0.00	0.12	62.18	60.47	1.72	0.21	0.02
Working spell: afternoon (%)	22.05	12.18	9.87	0.00	0.19	14.14	15.82	-1.69	0.10	-0.03
School track: academic (%)	55.48	58.89	-3.42	0.00	-0.05	53.97	54.27	-0.30	0.83	-0.00
School track: mixed (%)	23.91	18.84	5.07	0.00	0.09	20.62	21.90	-1.28	0.27	-0.02
School track: technical (%)	19.52	20.77	-1.25	0.18	-0.02	23.84	22.26	1.58	0.19	0.03
School track: pedagogic (%)	1.09	1.50	-0.41	0.11	-0.03	1.57	1.57	0.00	1.00	0.00
No. students	595.81	503.75	92.06	0.00	0.16	510.08	524.57	-14.49	0.18	-0.03
No. classrooms	15.93	14.76	1.17	0.00	0.09	14.42	14.57	-0.14	0.58	-0.01
Staff: No. principals	3.52	2.98	0.54	0.00	0.18	2.96	3.05	-0.10	0.08	-0.03
Staff: No. teachers	8.21	7.69	0.52	0.01	0.04	6.48	6.89	-0.40	0.07	-0.04
Staff: No. admin. support	21.18	20.40	0.79	0.01	0.04	19.17	19.47	-0.29	0.40	-0.02
Staff: No. healthcare support	0.15	0.23	-0.08	0.00	-0.10	0.15	0.14	0.01	0.58	0.01
Staff: No. psychological support	0.86	0.86	-0.00	0.91	-0.00	0.69	0.72	-0.04	0.22	-0.02
Teachers without college degree (%)	11.30	18.27	-6.97	0.00	-0.31	14.01	13.89	0.13	0.78	0.01
Panel B: Students socioeconomic composition from ICFES-SABER11 dataset										
Age at exam date	17.46	17.41	0.05	0.00	0.06	17.53	17.51	0.02	0.27	0.02
Students in school age (%)	81.86	83.07	-1.21	0.00	-0.06	80.01	80.45	-0.44	0.27	-0.02
Female students (%)	53.07	52.79	0.28	0.43	0.01	52.79	52.77	0.02	0.95	0.00
Ethnic min. students (%)	4.48	5.54	-1.07	0.01	-0.04	6.15	5.41	0.74	0.18	0.03
Working while studying (%)	4.49	3.59	0.90	0.00	0.06	4.30	4.40	-0.10	0.73	-0.01
Highly educated mother (%)	10.34	24.59	-14.25	0.00	-0.38	11.92	12.65	-0.73	0.24	-0.02
SES strata: very low (%)	42.36	38.56	3.80	0.00	0.07	50.76	47.96	2.80	0.01	0.05
SES strata: low (%)	42.19	33.74	8.45	0.00	0.18	35.17	36.43	-1.26	0.17	-0.03
SES strata: lower-middle (%)	14.09	20.71	-6.62	0.00	-0.18	12.34	13.77	-1.43	0.04	-0.04
SES strata: upper-middle (%)	1.09	4.67	-3.58	0.00	-0.27	1.37	1.46	-0.09	0.60	-0.01
SES strata: high (%)	0.19	1.57	-1.38	0.00	-0.21	0.26	0.27	-0.01	0.89	-0.00
SES strata: very high (%)	0.07	0.75	-0.68	0.00	-0.15	0.09	0.11	-0.02	0.59	-0.01
N. schools	3,760	3,727	7,487			2,483	2,484	4,967		

Source: Authors’ calculations based on SABER11 exam registers and DANE C-600 Census.

Regarding student characteristics, Panel B reports differences in the socioeconomic composition of students using information from the SABER11 exam dataset. On average, students from ethnic minorities have slightly lower representation in treated schools. Treated schools enroll a higher share of students from lower socioeconomic backgrounds, as measured by household income brackets, while control schools enroll fewer. In contrast, both groups of schools are similar in age distribution, gender composition, and the share of students engaged in child work while attending education.

Given the differences in attributes between treated and control schools, we use a nearest-neighbor propensity score matching procedure (NN-PSM) to obtain a subsample of comparable schools with better balance on observable characteristics, reduced selection bias, and a lower risk of violating the parallel-trends assumption in a difference-in-differences framework. Columns (6) to (10) report differences in means, p-values from traditional t-tests, and normalized differences following recent advances in the program-evaluation literature [19]. As expected, covariate balancing between treated and control schools improves in the matched sample, with a few remaining statistically significant differences likely due to chance; however, their economic relevance is minimal, as the corresponding normalized differences do not exceed 5 percent of an SD.

## 4. Empirical strategy

We evaluate the effects of increased school level retention on the secondary school exit exam performance at 11th grade. The empirical challenge in studying this question is that omitted variables may confound the relationship. A naive Ordinary Least Squares (OLS) estimation may be negatively biased for retained students if the lower scores they obtain are not due to retention but to their lower ability. As students’ ability increases, we might expect the benefits of grade retention to be decreasing and, for the upper end of the ability distribution, to negatively impact academic performance. Nonetheless, such counterproductive effects may be veiled by, for instance, the positive sorting of skilled students in subsequent grades as a byproduct of increased retention.

We exploit the before-mentioned policy change in Colombia that occurred in 2010 and implied that schools facing constraints in their retention requirements (AUP regime) were now allowed to retain as many students as they considered appropriate (FRP regime). To identify the effect of grade retention on test scores, we implement a difference-in-differences framework which exploits the school-year variation in retention rates.

The baseline two-way fixed effects (TWFE) specification we implement is:


$$Y_{st}=\alpha_{s}+\delta_{t}+\theta X_{st}+\beta \left[Treated_{s}*After_{t}\right]+\varepsilon_{st},$$ (1)

in which $Y_{st}$ denotes standardized test scores for school s in exam year t. $\alpha_{s}$ and $\delta_{t}$ are fixed effects by school and exam year, respectively. Parameter vector θ captures the influence of time-varying school’s characteristics $X_{st}$. $Treated_{s}$ is an indicator variable taking value of one if school s belongs to the treated group, and zero otherwise. It is important to remind that effects on test scores are only observable from 2011 onward since retention rates at 11th grade remained the same during the retention policy change. This implies that students taking the exam in 2010 were exposed to retention rates at grades 6th-10th under the AUP regime alone. Hence, the variable $After_{t}$ takes value one for all exam years affected by the AUP repeal (2011–2013). The parameter of interest is β, which captures the interaction between variables $Treated_{s}$ and $After_{t},$ and should be interpreted as the differential effect of increased retention on test scores between control and treated schools. Although the unit of observation corresponds to a school-exam year combination, robust standard errors are clustered at the municipality level to account for within-school serial correlation, and for the potential correlation between different schools within the same municipality, as suggested in the difference-in-differences literature [20].

The main identification assumption in this setting is that variation in retention rates is orthogonal to expected changes in test scores. Since it is impossible to test this assumption directly, we run an alternative specification to account for pre-reform trends in outcomes between treated and control schools. This assumption is equivalent to claim that both types of schools would have shared similar trends (not necessarily levels) in test scores if the retention policy had remained the same. We formulate an event-study (EE) specification to capture the dynamic effects of grade retention on test scores:


$$\begin{array}{c} \begin{array}{ccc} Y_{st} & = & \alpha_{s}+\delta_{t}+\sum_{k=2007}^{2009}\mu_{k}[Treated_{s}\times (Year=k)]\\ & & +\sum_{k=2011}^{2013}\beta_{k}[Treated_{s}\times (Year=k)]+\theta X_{st}+\varepsilon_{st}. \end{array} \end{array}$$ (2)

In equation (2), the null hypothesis of interest is that secular trend differences in tests scores during the AUP period between treated and control schools are not significantly different from zero (i.e., $\mu_{2007}=\mu_{2008}=\mu_{2009}=0$). We control for the interaction between the treatment status and those exam years where test takers, by construction, were not exposed to increased retention rates because of the policy change. In contrast, parameter $\beta_{k}$ captures the average effect of increased retention on tests scores at year k with k>2010. Given that traditional F-tests on the parallel trends assumption are underpowered, we resort to alternative tests available in the recent event-study literature [21,22], providing reassuring evidence that the causal effects obtained are robust to sizable deviations from the assumed common trend in test scores between treated and control schools.

Finally, we aim to analyze the effects of increased retention separately for retained and non-retained students by suggesting the following specification:


$$\begin{array}{c} Y_{st}=\alpha_{s}+\delta_{t}+\sum_{h=1}^{2}\gamma_{h}[Treated_{s}\times FRP_{t-h}]+\theta X_{st}+\varepsilon_{st}. \end{array}$$ (3)

$FRP_{t-h}$ is an indicator variable taking value 1 if the FRP regime was in place h *years before* exam year t, and zero otherwise. The interaction term $Treated_{s}\times FRP_{t-h}$ therefore measures the variation in tests scores that can be causally attributed to the shift in the retention policy from year t−h onward. While we do not observe individual retention outcomes, we can identify differential effects for retained and non-retained students by exploiting the variation in increased retention exposure by exam cohorts. The cohort of students taking the exam in year t is largely composed of two types of students: i) 10th grade students in year t−1 that were promoted to 11th grade at year t, and ii) 10th grade students that were retained in year t−2, repeated and passed 10th grade in year t−1 to subsequently be enrolled in 11th grade one year after. Hence, the pool of test-takers in exam cohorts 2011–2013 consists of non-retained students, and students who were retained in 10th grade only once. By controlling for the first two lags of our “FRP regime exposure” variable, we can differentiate the effect for each type of pupil.

In specification (3), our parameters of interest are $\gamma_{1}$ and $\gamma_{2}$. The first parameter measures the effect of being exposed one year to the FRP regime in 10th grade on schools’ tests scores in 11th grade one year after. The expected direction of this effect is ambiguous. On the one hand, the sign of $\gamma_{1}$ reveals whether non-retained students benefited from higher retention rates because of a positive sorting effect. In this case, we expect the effect to be positive. On the other hand, if we interpret this coefficient as the effect of increased retention for the marginal student (i.e., students that should have been retained but were promoted by a very small margin), we might expect the impact to have the opposite sign relative to the effect of retention on the retained students. For instance, if the latter effect is positive, non-retained students are worse off when promoted to 11th grade because they will miss the chance to receive further training on the academic subjects they struggled with the most. The second parameter $\gamma_{2}$ measures the impact of FRP regime’s exposure in the previous two consecutive years on schools’ test scores at year t. Assuming that students are retained in 10th grade only once, this impact can be mostly attributed to retained students (the second lag gives the effect on the retained and non-retained students minus the effect on the non-retained students).

Given our treatment-control classification, all coefficients of interests in the multiple specifications we described in this section can be best interpreted as intention-to-treat effects (ITT). To obtain the average treatment effects on the treated (ATT), it is possible to re-scale these coefficients by the difference in retention rates between treated and control schools implied by the law change.

## 5. Results

### The effect of the FRP regime on schools’ test scores

#### TWFE estimates.

Table 2 reports coefficients from a TWFE model as specified in equation (1). Estimates represent the average ITT effect of the AUP repeal on test scores during the entire FRP period. For average exam scores, which include all STEM and non-STEM subjects, treated schools scored 7.6 to 8.2 percent of an SD higher than comparable control schools after the repeal (columns (1) and (2)). Using exam percentiles as the performance measure, the repeal led to an average increase of up to 0.28 deciles, equivalent to a 5 percent rise relative to the median and 9 percent of as SD. Reported estimates do not differ by type of sample, as coefficients from the full and matched samples are statistically equivalent. Scaling these effects by the 6.5 p.p. increase in retention, this implies that one p.p increase in school retention leads, at least, to a 1.2% of an SD increase in tests scores.

**Table 2:** Effect of FRP regime on SABER11 test scores: TWFE estimates.

	Avg. Exam score		Exam percentile	
	(1)	(2)	(3)	(4)
Treated*After	0.076	0.082	2.469	2.761
	(0.007)***	(0.010)***	(0.223)***	(0.292)***
Matched sample	No	Yes	No	Yes
Mean (AUP period)	0.00	0.00	50.51	50.51
SD (AUP period)	1.00	1.00	28.81	28.81
Adj. R2	-0.211	-0.212	-0.212	-0.213
N. municipalities	564	549	564	549
N. Schools	7,487	4,967	7,487	4,967
Observations	42,242	27,770	42,242	27,770

Notes: This table presents estimates from the two way fixed effects (TWFE) model as described by equation (1). Columns (1) and (2) report results in which the dependent variable is the SABER11 standardized aggregate test score (all subjects). Columns (3) and (4) report results in which the dependent variable is the SABER11 percentile score. Columns (2) and (4) display results in which the nearest-neighbour propensity score matching (NN-PSM) subsample of schools is considered, while the remaining columns correspond to results in which the full sample of schools is used. Standard errors in parentheses are clustered at the municipality level. * p-value < 0.1, ** p-value < 0.05, *** p-value < 0.01.

**Fig 4:**
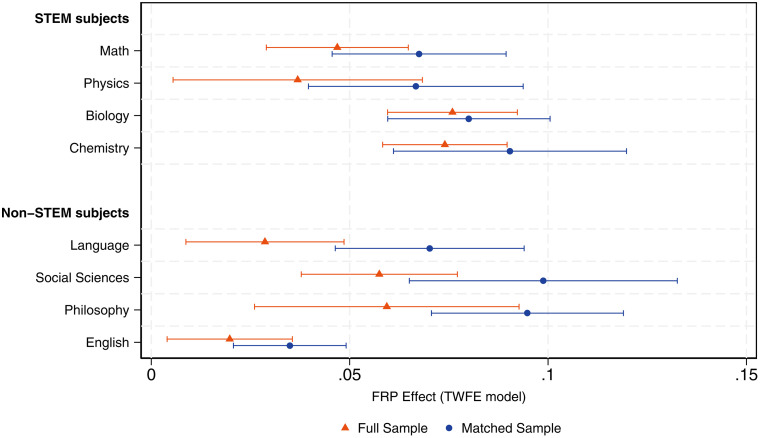
Effects of the FRP regime on average school test scores by academic subject. This figure displays TWFE point estimates on the effects of the FRP regime on SABER11 test scores by STEM and non-STEM academic subjects. Effects are measured in standard deviation units. Caps represent 95% confidence intervals.

To examine further effects of increased retention by academic subject, in Fig 4, we display TWFE estimates by STEM and non-STEM fields. On average, we observe that increased retention effects range between 2–8 percent of an SD. Considering estimates from the full sample, treated schools experienced gains in math and language of 5 and 3 percent of an SD, respectively. Biology and Chemistry exhibit the largest gains, with 8 and 7 percent of an SD effects in their respective scores. Non-STEM subjects also present sizable gains, with Philosophy being the academic subject that experienced the largest increase, on the order of 6 percent of an SD. When considering results from the matched sample of schools, effects are larger in magnitude, suggesting that by improving covariates balancing between treated and control schools, we can account for a potential downward bias in our causal effect estimate.

#### Event-study estimates.

**Fig 5:**
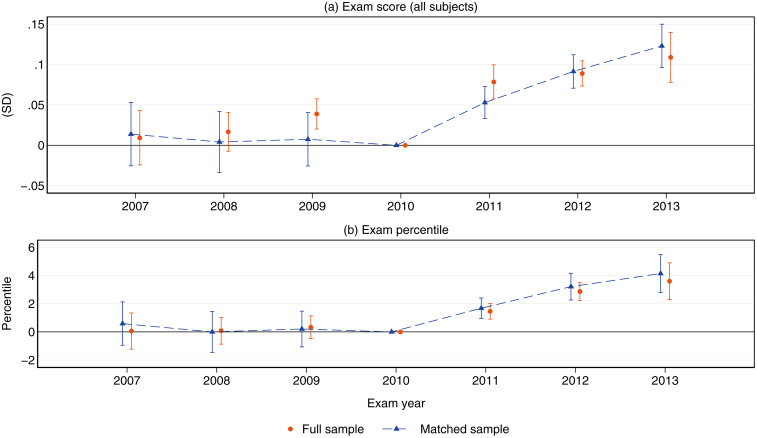
Effects of the FRP regime on average school test scores: Event-study estimates. This figure displays event-study point estimates on the effects of the FRP regime on SABER11 aggregate test scores (a) and performance percentiles gains **(b)**. Effects are measured in standard deviation units. Caps represent 95% confidence intervals.

To examine the dynamic evolution of the increased retention effect, Fig 5 displays the event-study estimates as implied by equation (2). Coefficients measure the average change in tests scores between treated and control schools in each year, setting the year 2010 as baseline. In general, estimates are fairly comparable between the full and matched samples. Relative to schools in the control group, treated schools increased their overall exam scores by 5.3%, 9% and 12% of an SD one, two, and three years after the AUP repeal, respectively (fig (a)). In terms of relative performance, treated schools scored 0.4 deciles higher in the exit exam than control schools. Importantly, coefficients associated with the AUP period are very close to zero in magnitude and statistically not significant. This finding hints that the parallel trends assumption holds. However, we provide several robustness checks to confirm that these results are robust to potential deviations from this assumption.

**Fig 6:**
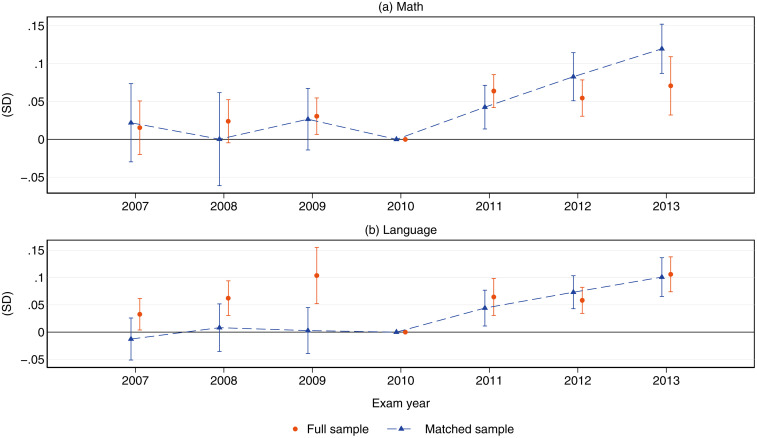
Effects of the FRP regime on average school test scores by subject: Event-study estimates. This figure displays event-study point estimates on the effects of the FRP regime on SABER11 math (a) and language (b) test scores. Effects are measured in standard deviation units. Caps represent 95% confidence intervals.

To contribute to the standardized test-scores literature, Fig 6 plots event-study coefficients for math and language scores. Gains in math following the AUP repeal are substantial, especially three years after the policy change, when treated schools show an additional 12% of an SD increase (Fig a). Language scores display similar patterns, with gains ranging from 4 to 10 percent of a standard deviation (Fig b). Compared with the overall exam-score results, the NN-PSM procedure clearly improves covariate balance between treated and control schools, removing several significant pre-trends that could undermine the parallel-trends assumption. For this reason, subsequent analyses rely on the matched sample of schools, yielding more consistent estimates with minimal loss of precision.

#### Plausible effects for retained and non-retained students.

Table 3 presents causal estimates on the effect of FRP exposure one and two years prior, according to equation (3). As previously discussed, the differential effect for treated schools when exposed to the AUP repeal one year before can be interpreted as the plausible effect of increased retention for non-retained students (first row). On average, non-retained students at treated schools scored an additional 7.4% of an SD in the SABER11 exam compared to students at control schools. These gains in performance are driven by both STEM and non-STEM subjects. For instance, math scores during the first lag of increased retention exposure rise 6.4% of a SD (column (2)). Language tests scores gains are statistically equivalent to those obtained in math (column (6)). In general, these findings suggest that non-retained students unambiguously benefited from increased retention, across all subjects.

**Table 3:** Effects of the FRP regime in Retained and Non-Retained Students.

	Exam Score	STEM				Non-STEM			
	(all subjects)	Math	Physics	Biology	Chemistry	Language	Philosophy	Social Sciences	English
	(1)	(2)	(3)	(4)	(5)	(6)	(7)	(8)	(9)
Treated*FRP (lag 1)	0.074	0.064	0.054	0.076	0.080	0.061	0.091	0.100	0.038
	(0.010)***	(0.012)***	(0.019)***	(0.012)***	(0.015)***	(0.013)***	(0.016)***	(0.014)***	(0.009)***
Treated*FRP (lag 2)	0.032	0.037	0.055	0.036	0.034	0.028	0.004	0.029	0.008
	(0.012)***	(0.013)***	(0.034)	(0.016)**	(0.016)**	(0.016)*	(0.018)	(0.015)*	(0.011)
Mean (AUP period)	0.00	0.00	0.00	0.00	0.00	0.00	0.00	0.00	-0.00
SD (AUP period)	1.00	1.00	1.00	1.00	1.00	1.00	1.00	1.00	1.00
Adj. R2	0.856	0.737	0.569	0.729	0.724	0.715	0.604	0.735	0.827
N. municipalities	549	549	549	549	549	549	549	549	549
N. Schools	4,967	4,967	4,967	4,967	4,967	4,967	4,967	4,967	4,967
Observations	27,770	27,770	27,770	27,770	27,770	27,770	27,770	27,770	27,770

Notes: This table presents estimates from the specification described in equation (3). First and second row estimates correspond to the interaction of the treatment status indicator variable with having being exposed to the free retention policy regime (FRP) one and two years before, respectively. Column (1) report results in which the dependent variable is the SABER11 aggregate standardized test score (all subjects). Columns (2) to (5) report results in which the dependent variables correspond to tests scores for subjects classified as part of the STEM field. Columns (6) to (9) present coefficients for tests scores in subjects classified as non-STEM. Standard errors in parentheses are clustered at the municipality level. * p-value < 0.1, ** p-value < 0.05, *** p-value < 0.01.

Effects attributable to retained students are more moderate (second row). On average, we observe that this group of students at treated schools scored 3% of an SD higher in the overall exam score, relative to their counterparts at control schools. This effect is roughly half of the magnitude reported for non-retained students. Regarding results by academic subjects, effects for STEM fields are salient for math, biology, and chemistry, in the order of 3% of an SD. While impacts on non-STEM subjects are smaller and most of them are not statistically significant, these estimates are still informative about rejecting negative effects of increased retention for plausibly retained students. For instance, 95% confidence intervals for language and social sciences tests scores indicate that we can reject negative effects as higher as 0.3% and 0.04% of an SD, respectively. When combining these two sets of findings, it is possible to conclude that the AUP repeal had an univocal positive impact on schools test scores, with non-retained students disproportionately benefiting more from increased school retention than their previously retained peers.

### Heterogeneous effects analyses

#### School attributes.

**Fig 7:**
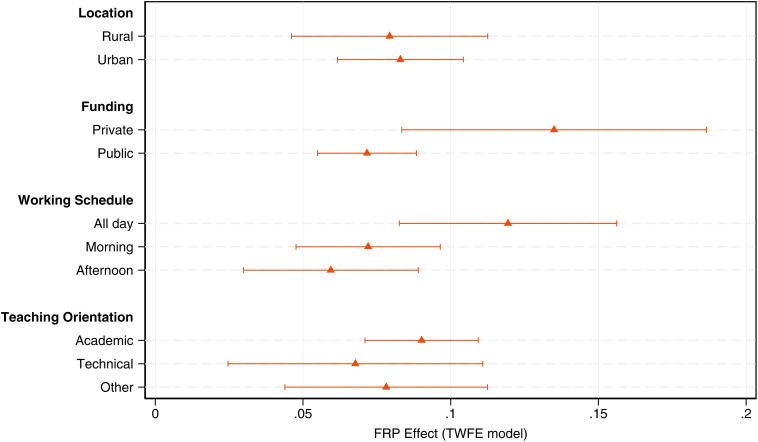
Heterogeneous effects of the FRP regime by school attributes. This figure displays TWFE point estimates on the effects of the FRP regime on aggregate SABER11 test scores (all subjects). Effects are measured in standard deviation units. Caps represent 95% confidence intervals.

In Fig 7, we plot the impact of the AUP repeal on exam scores by school attributes obtained from TWFE regressions. Urban and rural schools benefit similarly from increased retention, each showing gains of about 8 percent of a standard deviation. In contrast, heterogeneous effects are mediated by school funding type and day-shift schedule. On average, private schools exhibit a 13% of an SD increase in test scores, 6 p.p. higher than public schools in the treated group. Schools operating a full-day shift score, on average, 0.11 SD higher than control schools, and at least 4 p.p. higher than schools operating morning or afternoon shifts. Finally, we do not identify heterogeneous effects by teaching orientation, as estimates are similar across tracks and close in magnitude to those reported in Table 2.

#### Student characteristics.

**Fig 8:**
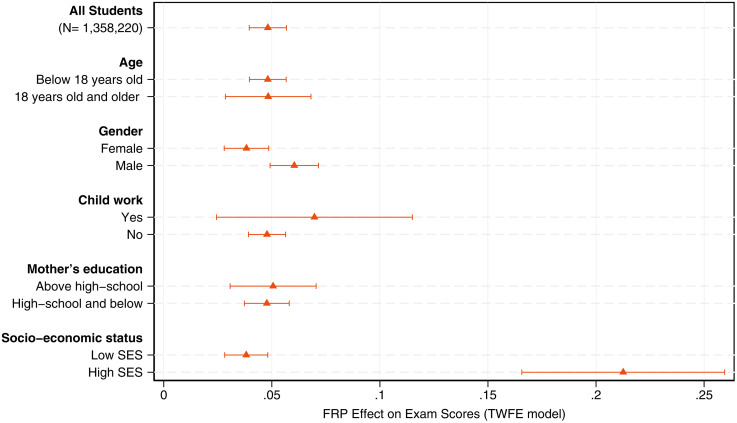
Heterogeneous effects of the FRP regime by student characteristics. This figure displays TWFE point estimates on the effects of the FRP regime on aggregate SABER11 test scores (all subjects). Effects are measured in standard deviation units. Caps represent 95% confidence intervals.

We display in Fig 8 the impact of increased retention on exam scores by differences in students characteristics. These estimates are obtained from TWFE models based on equation (1), now using students in the repeated cross-section dataset as the main unit of observation. Results indicate that retention effects for students are smaller in magnitude than the average effects identified at the school level. We do not find meaningful differences in outcomes by parental education (measured as the mother’s highest educational attainment) or by age group. There is a small difference in coefficients between students who engage in child work and those who do not (2% of an SD), but this estimate is imprecise.

In contrast, our findings show that individual heterogeneity in test scores gains following the AUP repeal is driven by gender and socioeconomic background. Male students experience a 6% SD increase in test scores, 3 p.p. higher than the gains observed for female students. Defining the group with low socioeconomic status (SES) as those families whose income lies below the median household income, results reveal a substantial gap in performance relative to students from more advantaged backgrounds. High-SES students score 0.2 SD -16 p.p. higher- than low-SES students in treated schools. Regardless of whether students were actually retained, the benefits of increased retention in secondary education are clearly assimilated more effectively by economically privileged students.

### Mechanisms

#### School quality.

Skeptics of social-promotion policies argue that students at the lower end of the ability distribution have weaker incentives to exert effort to be promoted. Thus, once the AUP regime is repealed, students at risk of being retained are expected to work harder, while effort provision and academic performance of students at the upper end of the distribution remain unaffected. To test this mechanism, we classify all schools in our matched sample into quartiles based on their average exam scores during the AUP years, and then estimate TWFE models separately for each quartile group. The dependent variable is the probability of belonging to a given exam-score quartile during the FRP period. The incentive argument would be supported if, for example, schools scoring in the first quartile (Q1) during the AUP period increased their probability of moving up to a higher quartile following the increased retention policy.

Table 4 presents FRP across the test-score distribution. Using results for all schools as a benchmark, schools at both the upper and lower tails of the distribution were the most responsive to the AUP repeal. On average, treated schools were 3 p.p. more (less) likely to perform at the last (first) quartile of the test score distribution than control schools. This effect is economically meaningful, corresponding to at least a 7% SD increase. Because i) low-performing schools are less likely to remain low performing after increasing retention, and ii) high-performing students are more likely to maintain strong performance, these findings suggest that the AUP repeal strengthened incentives to improve academic outcomes.

**Table 4:** Effects of the FRP regime along the AUP’s test scores distribution.

	SABER11 test scores			
	Q1	Q2	Q3	Q4
	(1)	(2)	(3)	(4)
All	-0.031	-0.009	0.009	0.032
	(0.007)***	(0.008)	(0.007)	(0.006)***
AUP mean	0.31	0.27	0.23	0.19
AUP SD	0.46	0.45	0.42	0.39
Adj. R-squared	-0.217	-0.218	-0.218	-0.216
N. municipalities	549	549	549	549
N. Schools	4,967	4,967	4,967	4,967
Observations	27,770	27,770	27,770	27,770
Q1 during AUP regime	-0.029	0.012	0.015	0.002
	(0.017)*	(0.016)	(0.008)*	(0.003)
Adj. R-squared	-0.215	-0.218	-0.211	-0.167
N. municipalities	373	373	373	373
N. Schools	1,606	1,606	1,606	1,606
Observations	8,577	8,577	8,577	8,577
Q2 during AUP regime	-0.055	0.003	0.041	0.012
	(0.017)***	(0.027)	(0.035)	(0.006)*
Adj. R-squared	-0.201	-0.194	-0.206	-0.200
N. municipalities	369	369	369	369
N. Schools	1,339	1.339	1.339	1.339
Observations	7.613	7.613	7.613	7.613
Q3 during AUP regime	-0.023	-0.040	0.004	0.059
	(0.009)***	(0.018)**	(0.027)	(0.015)***
Adj. R-squared	-0.189	-0.195	-0.187	-0.202
N. municipalities	328	328	328	328
N. Schools	1,148	1,148	1,148	1,148
Observations	6,579	6,579	6,579	6,579
Q4 during AUP regime	0.001	-0.017	-0.065	0.081
	(0.002)	(0.008)**	(0.016)***	(0.016)***
Adj. R-squared	-0.080	-0.188	-0.191	-0.191
N. municipalities	180	180	180	180
N. Schools	874	874	874	874
Observations	5,001	5,001	5,001	5,001

Notes: This table presents linear probability models estimates according to two way fixed effects (TWFE) specification described in equation (1). Each column presents results in which the dependent variable takes value one if the school’s SABER11 aggregate standardized test score reach a given quartile in the test scores distribution. First-row coefficients correspond to results in which all schools are considered. Coefficients displayed in rows 2–5 correspond to results in which schools are split by their average performance quartile during AUP years (2007–2009). Standard errors in parentheses are clustered at the municipality level. * p-value < 0.1, ** p-value < 0.05, *** p-value < 0.01.

When splitting the sample into performance quartiles during the AUP period, the previous conclusion becomes less clear-cut. The overall effect is driven by schools that performed above the median during the AUP years. Treated schools in the first and second quartiles are not significantly more likely to improve their performance during the FRP period than control schools with similar AUP-period performance. In contrast, treated schools in the third quartile (Q3) show a 6 p.p. higher probability of reaching the top quartile than comparable control schools. Top-performing schools (Q4) are 8 p.p. more likely to continue performing well in their respective quartile, and 6 p.p. less likely to fall into the third quartile. Taken together, these results indicate that the increased-retention reform strengthened incentives for above-median-performing students to exert additional effort.

#### School dropouts and transfers.

One potential concern is that the learning gains following the AUP repeal may be driven by (positive) selection into school enrollment. To test this channel, Table 5 reports a modified version of the TWFE model estimating the effect of the FRP regime on promotion, dropout, and transfer rates. In this analysis, we interact the treatment indicator with a binary variable equal to one for observations in the 2010–2013 period. This approach reflects the fact that, while impacts on test scores can only be identified one year after the repeal, changes in promotion, dropout, and transfer rates can be observed immediately once retention rates increase in 2010.

**Table 5:** Effects of the FRP policy regime on school promotion, dropouts, and transfers.

	School rates			Students		
	Promoted	Dropouts	Transferred	Promoted	Dropouts	Transferred
	(1)	(2)	(3)	(4)	(5)	(6)
Treated*After (2010)	-8.018	0.730	0.700	-47.515	2.901	4.892
	(0.149)***	(0.106)***	(0.100)***	(3.483)***	(0.490)***	(0.822)***
Mean (AUP period)	86.87	4.52	2.68	501.13	24.18	15.30
SD (AUP period)	8.76	4.80	3.88	373.14	30.95	26.46
Adj. R2	-0.094	-0.212	-0.212	-0.101	-0.214	-0.211
N. municipalities	564	564	564	564	564	564
N. Schools	7,487	7,487	7,487	7,487	7,487	7,487
Observations	42,224	42,224	42,224	42,224	42,224	42,224

Notes: This table presents estimates from the two way fixed effects (TWFE) model as described by equation (1). Columns (1) to (3) report results in which the dependent variable are promotion, dropout, and transfer rates per school. Columns (3) to (6) report results in which the dependent variable are the number of promoted, dropout and transferred students per school. Transferred students are define as those who changed schools during the academic year, while dropout students are defined as those who stopped attending education in their original school, without reappearing in the secondary school system registers later on. Standard errors in parentheses are clustered at the municipality level. * p-value < 0.1, ** p-value < 0.05, *** p-value < 0.01.

Promotion rates declined substantially after the AUP repeal. Treated schools experienced a 7.3 p.p. reduction in their average promotion rate relative to control schools, an 8 percent decrease compared with the average promotion rate before the FRP was implemented. Increases in dropout and transfer rates were more moderate. Transfers refer to students who move to a different academic institution, while dropouts are students who leave their original school without reappearing in the secondary education system. Relative to control schools, treated schools saw increases of 0.8 p.p. in dropout rates and 0.4 p.p. in transfer rates. These effects correspond to 14 percent and 10 percent of a standard deviation, respectively (columns (1) to (3)). Results using the number of students as the dependent variable (columns (4) to (6)) lead to similar conclusions.

Given the attrition of students, one concern is that the positive effects of the FRP regime are just a byproduct of students at the bottom of the ability distribution either dropping out from school or switching to schools with a more lenient approach. To address such caveat, we repeat the TWFE estimations, but we include dropout and transfer rates, and their respective interactions with the treatment indicator variable as additional covariates. The intuition behind this strategy is to test whether controlling for students’ attrition decreases the magnitude and statistical significance of the positive effects documented earlier. Results from this empirical exercise, presented in Table 6, confirm that the positive effects on tests scores cannot be attributed to students attrition alone. Irrespective of the specification considered, it is clear that effects are statistically equivalent in size, direction, and statistical significance to those initially reported (column (1)). Taken together, our findings indicate that while we document positive peer effects mostly attributed to non-retained students, the negligible influence of student attrition confirms that also plausibly retained students improve their learning outcomes, as reflected by their test scores gains.

**Table 6:** Effect of FRP regime on test scores: controlling for school dropouts and transfers.

	SABER11 Exam Score		
	(1)	(2)	(3)
Treated*After	0.0817	0.0822	0.0821
	(0.0104)***	(0.0104)***	(0.0110)***
Dropout rate		-0.0003	
		(0.0006)	
Transfer rate		-0.0017	
		(0.0007)**	
Treated*Dropout rate			0.0001
			(0.0009)
Treated*Transfer rate			-0.0008
			(0.0010)
Mean (AUP period)	0.00	0.00	0.00
SD (AUP period)	1.00	1.00	1.00
Adj. R2	-0.212	-0.212	-0.212
N. municipalities	549	549	549
N. Schools	4,967	4,967	4,967
Observations	27,770	27,770	27,770

Notes: This table presents estimates from the two way fixed effects (TWFE) model as described by equation (1). Column (1) displays baseline results as of Table 2. Columns (2) and (3) present extensions of the TWFE models in which dropout and retention rates, and their respective interactions with the treatment indicator variable, are included as additional covariates, respectively. Standard errors in parentheses are clustered at the municipality level. * p-value < 0.1, ** p-value < 0.05, *** p-value < 0.01.

## 6. Robustness checks

### Parallel trends assumption

The main identification assumption for considering our estimates as consistent approximations of the true causal effect is that, had the AUP regime continued, differences in test scores between treated and control schools would have evolved constantly across time. Our event-study estimates for the matched sample suggest that the parallel trend assumption holds, as traditional F-tests do not permit to reject the null hypothesis of diverging trends during the AUP period. To provide further convincing evidence of this fact, and taking into account that most pre-trend tests in a difference-in-differences setting seem to be underpowered, we implement two robustness checks that impose violations on the parallel trend assumption, assessing the validity of our findings under such violations.

Following the work of Roth (2022) [21], we first test how big a violation of the parallel trend in tests scores between treated and control schools would need to be, so it can be detected at least 80% of the time. S1 Fig displays point estimates and 95% robust-corrected confidence intervals from our event-study specification (2), corresponding to an 80% power, minimum detectable diverging trend of 0.02 SD (Likelihood Ratio = 0.002, Bayes Factor = 0.223). The dynamic effects under the assumption of this parallel trends violation are still statistically significant and sizable in magnitude. For instance, three years after the AUP repeal, test scores in treated schools improve an additional 7% of an SD, 5 p.p. less than the same effect reported under no diverging trends. Importantly, these results suggest that, in our setting, significantly higher diverging trends in outcomes can be identified by traditional tests from our event-study specification without need to worry about test power. Worst case scenario, our results are attenuated but are still sizable in magnitude and statistical relevance.

In the second robustness check, we implement the methodology suggested by Rambachan and Roth (2023) [22] to assess the extent to which our reported effects are still statistically significant, even when major deviations from the common trend in test scores shared by treated and control schools are allowed. S2 Fig present the plots of the different effect bounds on aggregate test scores under relative magnitude changes in trends before and after the AUP repeal. Findings from this test suggest that our setting survives sizable deviations from the parallel trend assumption. In particular, results remain significant even if the post-treatment violation of parallel trends is 1.2 SD higher than the pre-trend associated to the AUP period. Even if such deviation is in the order of 1.4 SD, effect bounds allow us to reject negative effects as large as 0.6% of an SD. All these results combined indicate that the parallel trend assumption in our difference-in-differences setting is appropriate.

### Other robustness checks

S3 Fig presents point estimates and 95% confidence intervals from TWFE models, only considering the balanced sample of schools (N. schools = 1,917; 13,419 observations), as an attempt to understand whether our findings are robust to the potential attrition of some schools from our sample because of market reasons (i.e., schools operating only in some years of our study period). We also plot estimates from TWFE models that correspond to the matched sample of schools as a comparison benchmark. We can confidently reject any potential concern with regards to school selective attrition, as all estimates are statistically equivalent irrespective of the sample considered.

A potential concern with regards to our empirical strategy is that our results are driven by the presence of retention outliers, i.e., schools that exhibited abnormally higher retention rates, close to 100%, or schools that presented zero, or even negative retention rates after the AUP repeal. S4 Fig presents estimates in which we compare those TFWE coefficients from the matched sample of schools (our benchmark), with estimates in which we dropped schools at the top and bottom 5% tails of the retention distribution. Results coincide in magnitude and precision, suggesting that results in our setting are not driven by abnormal differences in retention rates by outlier schools.

Finally, we consider whether our results are robust to defining alternative treatment classifications. It is worth to remember that we defined treated schools as those exhibiting an above-median change in retention rates between the AUP and FRP periods. S1 Table presents results in which we define treated schools as those with above-median retention levels during AUP years. Considering retention rates at the school level (first row), it can be observed that results remain significant, although magnitudes are attenuated. For instance, treated schools increased their test scores by 0.3% SD relative to control schools, which is at least 4 p.p. lower than the effect reported in our principal results (Table 2). Effects are more pronounced for STEM subjects (between 3% to 5% of an SD), with impacts for non-STEM subjects becoming imprecise and even negative. When considering retention rate levels for grades 6th to 8th, and 9th to 11th, it is evident that most of the variation comes from retention at the upper secondary education stage. Treated schools, on average, scored 4% of an SD higher in the SABER11 exam than control schools. Overall, our findings are robust to this alternative treatment-control school classification, the estimates of which can be regarded as a lower bound of the true effect of increased retention on test scores.

## 7. Concluding remarks

This paper analyzes the effect of school retention on 11th-grade school performance. We exploit a policy change in Colombia that altered retention rules. Until 2010, schools were allowed to retain no more than 5 percent of their students. After the law was repealed in 2010, schools could retain as many students as they deemed appropriate. This shift produced a large increase in grade retention, with substantial variation across schools. Using a difference-in-differences strategy, we estimate the impact of such policy change on performance at the high-stakes, high-school exit standardized exam. Multiple robustness checks support the validity of our findings, even when allowing for sizable deviations from the parallel-trends assumption among schools that responded differently to the policy change.

Our estimates reveal that all students unambiguously benefited from increased school retention. These benefits are heterogeneous. Retained students, female students, students from low SES households, students enrolled in schools at the bottom of the performance distribution prior to the policy change, and students enrolled in public schools, are identified as the groups that benefited the least from the AUP repeal. By contrast, positively selected students obtained the largest gains, a result explained by a simultaneous rise in school dropouts and transfers, and the empowering of incentives to perform better at those schools at the top of the ability distribution. We reject the strategic substitution of effort between STEM and non-STEM subjects as our results show improvement in both areas.

This research shows the importance of analyzing effects of retention at different margins of the ability distribution and across different socioeconomic divides. Although data restrictions do not allow to recover information on individual retention, we feel confident that the empirical strategy and data construction implemented in this paper aids to solve this limitation by decomposing the effect of retention among different types of students. More research is needed to investigate whether the gains of retention we identify can be outweighed by other costs of retention, such as school dropouts, career choice regret, delayed (or sudden) labor market participation, forgone income, and the formation of undesirable personality traits, preferences and risk attitudes across the life cycle.

## Supporting information

S1 Fig Roth (2022) pre-trends sensitivity analysis. This figure displays coefficients from an event-study specification, as well as corrected point estimates assuming a minimum diverging trend between treated and control schools that can be detected 80% of the time. Caps represent 95% confidence intervals. The dotted line show the corrected point estimates. The continuous line shows the hypothesized trend. Effects are measured in standard deviation units.
(EPS)

S2 Fig Rambachan and Roth (2023) pre-trends sensitivity analysis. This figure shows different effect bounds on aggregate SABER11 test scores under relative magnitude changes in trends before and after the AUP repeal. Caps represent confidence intervals. Effects are measured in standard deviation units.
(EPS)

S3 Fig Robustness checks: controlling for school attrition. This figure displays TWFE point estimates on the effects of the FRP regime on aggregate SABER11 test scores for the balanced panel of schools. Effects are measured in standard deviation units. Caps represent 95% confidence intervals.
(EPS)

S4 Fig Robustness checks: controlling for school outliers. This figure displays TWFE point estimates on the effects of the FRP regime on aggregate SABER11 test scores, while dropping the top and bottom 5% of the retention rate change distribution. Effects are measured in standard deviation units. Caps represent 95% confidence intervals.
(EPS)

S1 Table Effect of FRP regime on test scores: Alternative treatment group definitions. Notes: This table presents estimates from the two way fixed effects (TWFE) model as described by equation (1). Rows report coefficients that correspond to the interaction of alternative treatment-control classifications with a time indicator variable for the FRP period. Treated schools are defined as those with above-median retention rates levels during the AUP period (2007–2009). All specifications include fixed effects at the school and year levels. Standard errors in parentheses are clustered at the municipality level. * p-value < 0.1, ** p-value < 0.05, *** p-value < 0.01.
(RTF)
