# Supplementary material for: The effect of grade retention on secondary school performance: Evidence from a natural experiment
Source: PLoS One. 2026 Mar 17;21(3):e0345322. doi: 10.1371/journal.pone.0345322 (PMC12994823; doi:10.1371/journal.pone.0345322)
Supplement: S1 Table — Notes: This table presents estimates from the two way fixed effects (TWFE) model as described by equation (1). Rows report coefficients that correspond to the interaction of alternative treatment-control classifications with a time indicator variable for the FRP period. Treated schools are defined as those with above-median retention rates levels during the AUP period (2007–2009). All specifications include fixed effects at the school and year levels. Standard errors in parentheses are clustered at the municipality level. * p-value < 0.1, ** p-value < 0.05, *** p-value < 0.01. (RTF) [file pone.0345322.s005.rtf]

 Table S1: Effect of FRP regime on test scores: Alternative treatment group definitions
Above-median	Total	STEM	Non-STEM	
Retention level	Exam	Math	Physics	Biology	Chemistry	Language	Philosophy	Soc. Sciences	English	
*After	(1)	(2)	(3)	(4)	(5)	(6)	(7)	(8)	(9)	
School	0.025	0.033	0.054	0.025	0.030	0.009	0.023	0.016	-0.003	
	(0.014)*	(0.014)**	(0.019)***	(0.013)**	(0.015)*	(0.017)	(0.021)	(0.020)	(0.013)	
Adj. R2	0.862 	0.752 	0.583 	0.731 	0.733 	0.710 	0.607 	0.737 	0.841 	
N. municipalities	527 	527 	527 	527 	527 	527 	527 	527 	527 	
N. Schools	4,402 	4,402 	4,402 	4,402 	4,402 	4,402 	4,402 	4,402 	4,402 	
Observations	24,468	24,468	24,468	24,468	24,468	24,468	24,468	24,468	24,468	
										
6th to 8th grade	0.024	0.014	0.031	0.030	0.029	0.037	0.063	0.016	0.009	
	(0.014)*	(0.012)	(0.015)**	(0.015)**	(0.017)*	(0.020)*	(0.022)***	(0.027)	(0.010)	
Adj. R2	0.863 	0.753 	0.586 	0.732 	0.736 	0.711 	0.610 	0.737 	0.842 	
N. municipalities	526 	526 	526 	526 	526 	526 	526 	526 	526 	
N. Schools	4,219 	4,219 	4,219 	4,219 	4,219 	4,219 	4,219 	4,219 	4,219 	
Observations	23,559	23,559	23,559	23,559	23,559	23,559	23,559	23,559	23,559	
										
9th to 11th grade	0.042	0.028	0.056	0.059	0.050	0.060	0.093	0.048	0.018	
	(0.010)***	(0.014)**	(0.016)***	(0.012)***	(0.014)***	(0.013)***	(0.021)***	(0.016)***	(0.008)**	
Adj. R2	0.863 	0.752 	0.584 	0.732 	0.736 	0.712 	0.610 	0.737 	0.843 	
N. municipalities	527 	527 	527 	527 	527 	527 	527 	527 	527 	
N. Schools	4,199 	4,199 	4,199 	4,199 	4,199 	4,199 	4,199 	4,199 	4,199 	
Observations	23,474	23,474	23,474	23,474	23,474	23,474	23,474	23,474	23,474	
Notes: This table presents estimates from the two way fixed effects (TWFE) model as described by equation (1). Rows report coefficients that correspond to the interaction of alternative treatment-control classifications with a time indicator variable for the FRP period. Treated schools are defined as those with above-median retention rates levels during the AUP period (2007-2009). All specifications include fixed effects at the school and year levels. Standard errors in parentheses are clustered at the municipality level. * p-value < 0.1, ** p-value < 0.05, *** p-value < 0.01.
